# High-Throughput Metabolomics Integrated Network Pharmacology Reveals the Underlying Mechanism of Paeoniae Radix Alba Treating Rheumatoid Arthritis

**DOI:** 10.3390/molecules27207014

**Published:** 2022-10-18

**Authors:** Lei Liu, Taiping Li, Hui Dong, Xijun Wang

**Affiliations:** 1 GAP Research Center of Chinese Medicine, Chinmedomics Research Center of State Administration of TCM, Heilongjiang University of Chinese Medicine, Harbin 150040, China; 2 School of Parmacy and Pharmaceutical Sciences & Institute of Materia Medica, Shandong First Medical University & Shandong Academy of Medical Sciences, Jinan 250100, China

**Keywords:** *Paeoniae Radix Alba*, rheumatoid arthritis, metabolomics, paeoniflorin, kaempferol, amino acid metabolism

## Abstract

Objective: The mechanism of action and potential targets of *Paeoniae Radix* *Alba* (Baishao, B) in the treatment of adjuvant-induced arthritis (AIA) rats are explained using metabolomics and network pharmacology techniques, and the research evidence for the development of anti-rheumatoid arthritis (RA) drugs is enriched. Methods: The rats were injected with Freund’s complete adjuvant (CFA) to induce arthritis. We then measured the general physical characteristics, examined their X-rays and histopathology to evaluate the pathological condition of the inflammation models, and conducted metabolomics studies on the change in urine metabolism caused by CFA. The lyophilized powder of B at a dose of 2.16 g/kg was orally administered to the rats continuously for 28 days, and the therapeutic effect was evaluated. Network pharmacology prediction shows that B contains the target action of the ingredient, and the simulation of the target molecular docking, in combination with the metabolomics analysis results, shows that B has a potential role in the treatment of AIA rats. Results: B can reduce the paw swelling and pathological changes in rats caused by CFA, reverse the levels of 12 urine biomarkers, and regulate histidine metabolism, phenylalanine metabolism, arginine, proline metabolism, pyrimidine metabolism, etc. The prediction of the active ingredient target in B indicates that it may act as an inflammatory signaling pathway in anti-RA, among them being paeoniflorin, palbinone, beta-sitosterol, kaempferol, and catechin, which are the significant active ingredients. Conclusion: The metabolomics results revealed the markers and metabolic mechanisms of urinary metabolic disorders in rats with AIA, demonstrated the efficacy of the therapeutic effect of B, and identified the key ingredients in B, providing theoretical support for the subsequent development and utilization of B.

## 1. Introduction

*Paeoniae radix alba* (Bai Shao, B) is the dried root of *Paeoniae lactiflora* Pall. The total glucosides of the paeony of terpenes, such as paeoniflorin, albiflorin, oxypaeoniflorin, benzoylpaeoniflorin, and benzoyloxypeoniflorin, are the major chemical ingredients of B. B is a valuable traditional medicine that is used to cure liver disease, rheumatism, and autoimmune diseases [1,2,3,4]. Numerous in vivo and in vitro reports on the pharmacological activity and mode of action of B and its extracts are currently available. As an example, B polysaccharides derived through improved extraction can successfully treat experimental autoimmune hepatitis [5], and B’s active ingredient decreases the damaging reaction of nonalcoholic fatty liver disease [6]. B and its extracts play a vital role in the treatment of immune diseases. First, in rats with arthritis, B and its extracts can limit the proliferation of fibroblast synoviocytes and lower the synovial inflammatory response [7]. Additionally, the regulation of immune cells is a key function of B and its extracts in the treatment of immunological illnesses. B and its extracts suppress dendritic cell maturation and activation, which impairs Th1 and Th17 differentiation in vivo and drastically lowers the proportion and total number of Th1 and Th17 cells in collagen-induced arthritic mice [8]. Furthermore, the latest information indicates that the terpenoid active components in B significantly reduce rheumatoid arthritis (RA), with palbinone being confirmed to be an inhibitor responsive to crucial signals [9]. In conclusion, B and its extracts profoundly regulate immunological abnormal disorders, particularly the complicated treatment of arthritis.

Metabolomics technology with mass spectrometry as a tool has been valued and applied in the pharmacological efficacy research of traditional Chinese medicine and natural products. Network pharmacology further realized the huge chemical compositions in herbs for disease resistance and the treatment of multiple-target, multiple-pathway mechanism predictions for the biological activity of herbal natural products. In the face of huge, complex chemical compositions, mass spectrometry with a high sensitivity to identifying compounds expounds the molecular markers and metabolic mechanism of disease [10,11,12,13].

**Figure 1:**
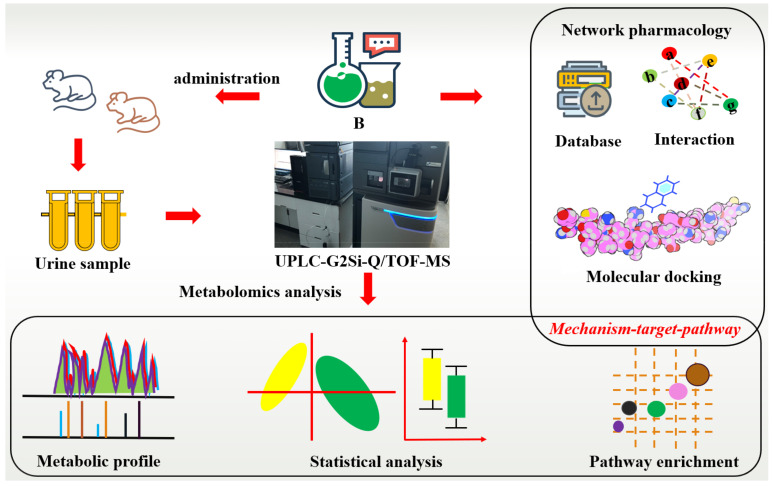
Flow chart of mass spectrometry-based metabolomics combined with network pharmacological analysis of the mechanism of action of *Paeoniae Radix Alba* in rheumatoid arthritis.

This study will evaluate the effectiveness of B for RA based on high-resolution mass spectrometry, analyze the ability of B to regulate abnormal metabolism effectively, and reveal the potential active ingredients of B for RA using network pharmacology techniques. It aims to supplement the evidence for the mechanism of anti-RA action in B and promote the development of promising compounds of B (Figure 1).

## 2. Materials and Methods

### 2.1. Drugs and Reagents

The B was purchased from the Tongrentang Drug Store and authenticated by Professor Xijun Wang of the Department of Pharmacognosy of Heilongjiang University of Chinese Medicine (Harbin, China). High-performance liquid chromatography-grade methanol and acetonitrile were purchased from Thermo Fisher (Waltham, MA, USA), the leucine enkephalin and Freund’s complete adjuvant (CFA) were purchased from Sigma Aldrich (Darmstadt, Germany), and the distilled water was purchased from Watsons (Guangzhou, China). In this study, B was decocted in 10 volumes of ultrapure water for 30 min (twice) and then prepared into a freeze-dried powder and dissolved in appropriate water to form a dosing solution.

### 2.2. Drug Administration

The Changsheng Animal Research Center (Liaoning, China) provided the 8-week-old male Sprague Dawley rats for the experiment. The rats were adapted to being raised for 7 days in a stable environment (12:12-h light/dark schedule; temperature: 232 °C; relative humidity: 40–60%) with unlimited access to food (common grade feed: corn, wheat bran, soybean meal, grain flour, salt, etc.) and water. Twenty-four rats were randomly divided into a control group, model group, and treatment group (B). To create RA-related pathological models, the rats were injected with 0.1 mL of CFA at a concentration of 2.0 mg/mL. The control group received an equal volume of saline as an injection [14]. On the 14th day, the rats in group B were given a 2.16-g/kg B freeze-dried powder aqueous solution for 28 days, and the other rats were given an equal volume of distilled water.

### 2.3. Evaluation of Swelling and Pathological Characteristics

To assess the pathological status of adjuvant-induced arthritis (AIA) during the study period, the claw volume for each group of rats was measured, and the arthritis index (AI) was calculated [15]. The rats were sacrificed on the 28th day of B administration for ankle X-ray observation, joint synovium analysis, hematoxylin and eosin (H&E) examination, and organ index calculation to evaluate B’s therapeutic effect.

### 2.4. Urine Sample Collection and Preparation

On day 28, we collected urine from the AIA rats to identify the biomarkers and monitor metabolism for assessing the treatment’s impact. The urine from the stage of 12 h was collected from all rats between the hours of 8:00 p.m. and 8:00 a.m. the following day in separate metabolic cages. The urine samples were centrifuged for 10 min at 4 °C and at 13,000 rpm. The supernatant was aspirated, filtered with a 0.22-μm filter membrane, and analyzed by ultra-performance liquid chromatography-quadrupole time of flight mass spectrometry (UPLC-Q/TOF-MS, Waters, Milford, MA, USA).

### 2.5. Metabolomics Analysis Conditions

The chromatography was equipped with an ACQUITY™ UPLC system and separated in an HSS T_3_ column (100 mm × 2.1 mm id, 1.8 µm; Waters, Milford, MA, USA), where the column temperature was 35 °C, the flow rate was 0.4 mL/min, and the injection volume was 2 μL. Acetonitrile with 0.1% formic acid (A) and water with 0.1% formic acid (B) were the mobile phases. The optimized gradient elution conditions were 0–4.5 min and 1–21% A, 4.5–7 min and 21–40% A, 7–7.5 min and 40–50% A, 7.5–9.5 min and 50–99% A, 9.5–10 min and 99–99% A, and 10–14.5 min and 99–1% A.

The MS was equipped with an electrospray ion source. In positive ion mode, the capillary voltage was 3 kV, the cone voltage was 20 V, the ion source temperature was 110 °C, the dissolvent gas temperature was 350 °C, and the flow rate was 800 L/h. The cone gas flow rate was 50 L/h, a capillary voltage in negative ion mode was 2.7 kV, and other analysis conditions were the same as for positive ion mode. Leucine enkephalin with a concentration of 1 ng/μL and a flow rate of 10 μL/min was used for real-time mass correction in positive mode ([M + H]^+^ = 556.2771) and negative mode ([M − H]^−^ = 554.2615), with centroid mode at a 50–1200-m/z full scan to collect data.

### 2.6. Data Processing and Multivariate Data Analysis

The raw data were acquired by UPLC-MS using Progenesis QI 2.0 software (Waters, Milford, MA, USA) to perform peak noise reduction, matching, normalization, and alignment to obtain a matrix with the retention time, molecular formula, normalized abundance, and m/z, SIMCA-P 14.0 for principal ingredient analysis (PCA), and orthogonal partial least squares discriminant analysis (OPLS-DA) to analyze the differences between groups and determine the variable importance in projection value (VIP) (>1), combined with the student’s *t*-test statistics (*p* < 0.05) difference ion for online database biological function analysis (HMDB, http://www.hmdb.ca/, accessed on 19 November 2020; METLIN, http://www.metlin.scripps.edu/, accessed on 19 November 2020; and Lipid Maps, http://www.lipidmaps.org/, accessed on 19 November 2020), and mass tolerances <10 ppm, and those with MS/MS fragmentation information were considered to be important biomarkers.

### 2.7. Network Pharmacology and Molecular Docking Analysis Methods

B contains a compound information query from the traditional Chinese medicine systems pharmacology database and analysis platform (TCMSP, http://lsp.nwsuaf.edu.cn/index.php/, accessed on 20 November 2020). We selected the compounds with the pharmacokinetics parameters of oral bioavailability (OB) ≥30% and drug-likeness (DL) ≥0.18. We also utilized GeneCards (https://www.genecards.org/, accessed on 20 November 2020) and DisGeNET (https://www.disgenet.org/home/, accessed on 20 November 2020) to retrieve the targets associated with RA and align them to those of the selected compounds described above for further identifying potentially acting RA targets for pathway analysis and molecular docking studies. Visualization of ingredient-target-pathway interaction was achieved using Cytoscape 3.8.0 software [16], while Autodock 1.5.6 software was used for molecular interaction pattern prediction and a molecular docking approach for exploring the binding modes of the key compounds [17].

## 3. Results

### 3.1. Inhibitory Effect of B on Rat AIA Paw Swelling

**Figure 2:**
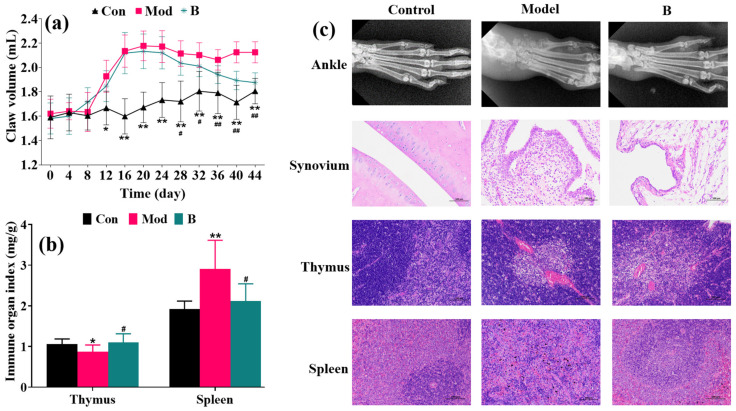
The physical signs measured during model preparation, showing the results of histological pathology (×200) and X-ray examination after 28 days of oral administration of B. (**a**) Claw volume. (**b**) Indexes of the spleen and thymus. (**c**) X-ray and histological pathology. * *p* < 0.05. ** *p* < 0.01 vs. control group. ^#^ *p* < 0.05. ^##^ *p* < 0.01 vs. model group.

Compared with the control group, the paw volume of the rats in the model group after injection of CFA was significantly larger on days 12–16 (Figure 2a). As a vital immune organ, the thymus of the model group was atrophied, and the spleen was swollen (Figure 2b). In addition, the X-ray examination results showed stenosis of the ankle joint space and soft tissue edema in the rats’ model group. The model group’s synovium, spleen, and thymus tissues displayed inflammatory pathological alterations according to the results of the histological analysis. However, B was able to endure this condition after 28 days of treatment (Figure 2c).

### 3.2. Multivariate Statistical Analysis and Biomarker Characterization of the Metabolic Profile

**Figure 3:**
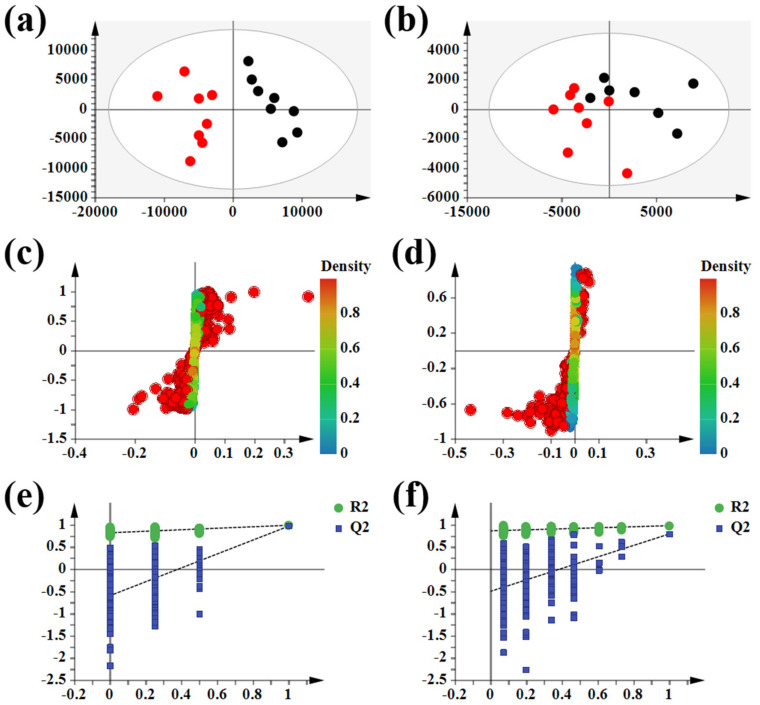
Multivariate statistical analysis of control groups and model groups. Note: (●) = control group and (●) = model group. (**a**) PCA scores plotted in positive mode. (**b**) PCA scores plotted in negative mode. (**c**) S-plot in positive mode. (**d**) S-plot in negative mode. (**e**) Permutation plot in positive mode. (**f**) Permutation plot in negative mode.

On day 14, the urine metabolic profiles of the model group and the control group were subjected to PCA pattern recognition (Figure 3a,b). The model group’s metabolism changed as a result of the CFA injection because there were distinct differences between the control and model groups. OPLS-DA was employed to screen potential biomarkers, and the R2Y and Q2 values of the model (R2Y(cum) = 0.995, Q2 (cum) = 0.974 in positive mode and R2Y(cum) = 0.985, Q2 (cum) = 0.799 in negative mode) indicated that the model had good quality and predictability. An S-plot was used to screen for important metabolic difference ions where VIP > 1 (Figure 3c,d), and the results of the permutation prediction (*n* = 200) showed that the OPLS statistical model did not overfit (Figure 3e,f). A Student’s *t*-test was applied to analyze the difference ions where VIP > 1 and the normalized abundance was statistically significant (*p* < 0.05). These important difference ions were considered potential biomarkers, combining MS and MS/MS structural information with HMDB and KEGG biological function analysis, including betaine, N2-succinyl-L-ornithine, phenyl pyruvic acid, pyruvic acid, and taurine. Finally, 29 potential biomarkers were found to be closely related to AIA (Table S1).

### 3.3. Metabolic Pathway Integration Enrichment Analysis

**Figure 4:**
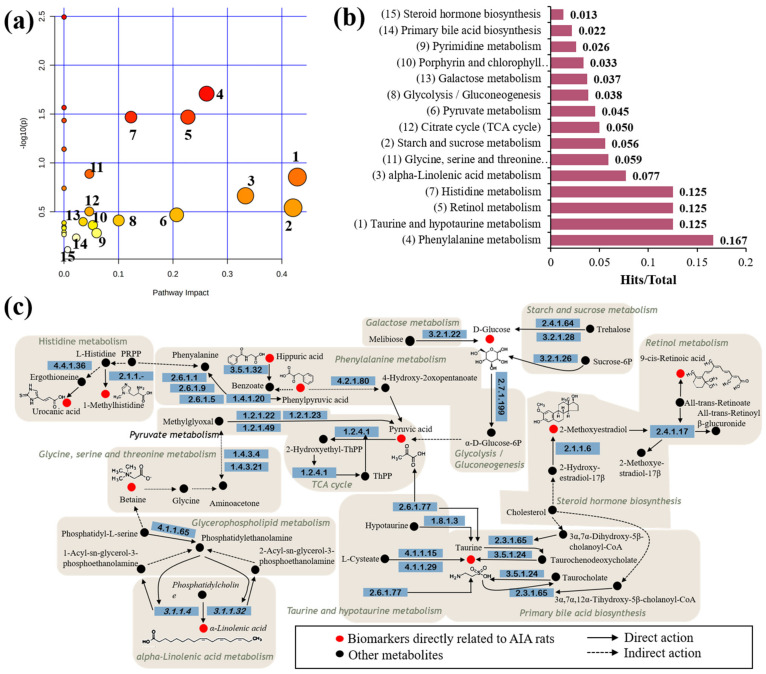
Enrichment analysis of MetPA metabolic pathway of biomarkers. (**a**) Pathway impact enrichment. (1) Taurine and hypotaurine metabolism. (2) Starch and sucrose metabolism. (3) Alpha-linolenic acid metabolism. (4) Phenylalanine metabolism. (5) Retinol metabolism. (6) Pyruvate metabolism. (7) Histidine metabolism. (8) Glycolysis or gluconeogenesis. (9) Pyrimidine metabolism. (10) Porphyrin and chlorophyll metabolism. (11) Glycine, serine, and threonine metabolism. (12) Citrate cycle (TCA cycle). (13) Galactose metabolism. (14) Primary bile acid biosynthesis. (15) Steroid hormone biosynthesis. (**b**) The proportion of biomarkers in the overall pathway. (**c**) Association network of biomarker metabolic pathways.

The above 29 potential biomarkers were subjected to MetaboAnalyst (https://www.metaboanalyst.ca/, accessed on 26 November 2020) metabolic pathway enrichment analysis to reveal the mechanism of the metabolic differences in the rats after receiving CFA injections. The results showed that these biomarkers were involved in multiple metabolic pathways, such as taurine and hypotaurine metabolism, starch and sucrose metabolism, alpha-linolenic acid metabolism, phenylalanine metabolism, and glycolysis or gluconeogenesis, which together lead to the development of inflammatory pathological conditions (Figure 4a–c).

### 3.4. B Regulates Biomarkers and Metabolic Trajectories to Play a Therapeutic Role

**Figure 5:**
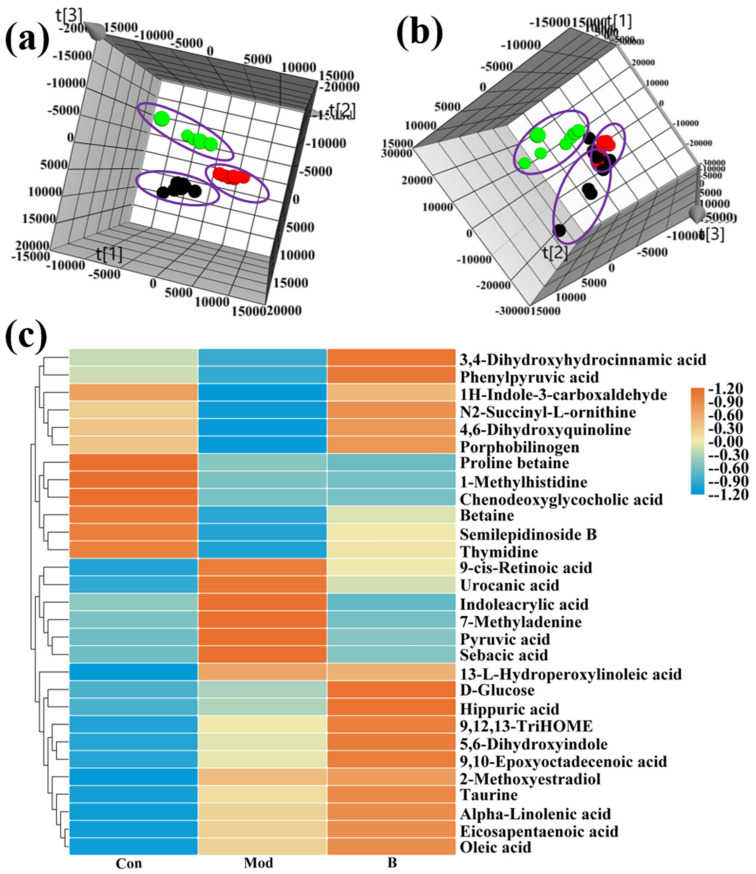
Metabolic regulation and cluster analysis of biomarkers in B treatment of AIA rats. Note: (●) = control group, (●) = model group, and (●) = Treatment group (B). (**a**) The 3D PCA scores plotted in positive mode. (**b**) The 3D PCA scores plotted in negative mode. (**c**) Euclidean Cluster analysis of biomarker levels.

**Figure 6:**
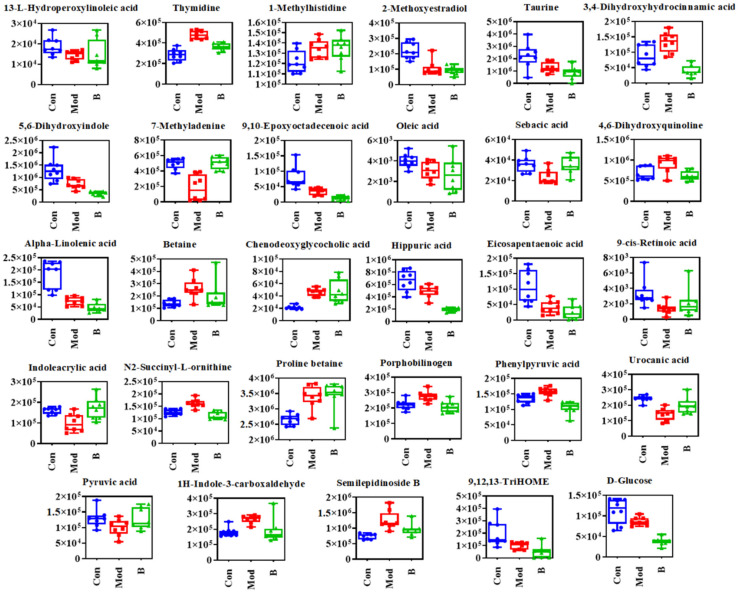
Box plots of the normalized abundance of biomarkers in the control group, model group, and treatment group (B).

The PCA results after 28 days of treatment demonstrate that group B was between the control group and the model group, with a propensity to shift to the control group in the case where B could resist the pathological alterations generated by CFA in rats (Figure 5a,b). The three groups of Euclidean cluster analysis also showed that B reversed the normalized abundance level of metabolites (Figure 5c), making the levels of many biomarkers closer to the control group (Figure 6), including 1H-indole-3-carboxaldehyde, 4,6-dihydroxyquinoline, 7-methyladenine, lndoleacrylic acid, N2-succinyl-l-ornithine, phenyl pyruvic acid, porphobilinogen, semilepidinoside B, thymidine, urocanic acid, 3,4-dihydroxyhydrocinnamic acid, and sepacic acid.

### 3.5. Predicting the Target and Binding Mode of B

**Figure 7:**
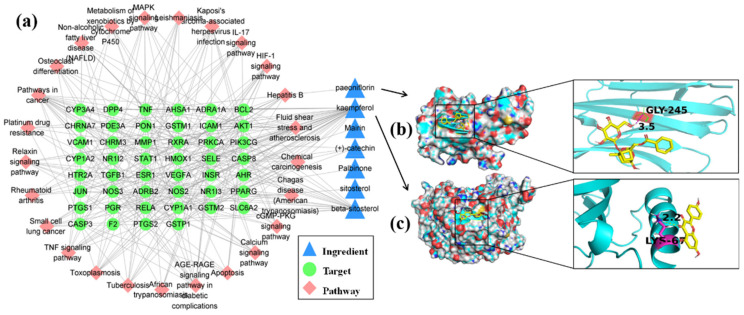
Network pharmacological target prediction and molecular docking simulation of active ingredients contained in B. (**a**) Ingredient-target-pathway network. (**b**) The binding mode of the representative ingredient paeoniflorin and the target TNF. (**c**) The binding mode of the significant ingredient kaempferol and the target AKT.

Network pharmacology is currently widely used in Chinese medicine research to predict the targets and potential mechanisms of herbs and prescriptions. The ingredients and targets with the pharmacokinetic parameters OB ≥ 30 and DL ≥ 0.18 were searched for in the TCMSP database for string (https://string-db.org/cgi/input.pl/, accessed on 20 November 2020) KEGG pathway analysis, and it was found that palbinone, paeoniflorin, mairin, beta-sitosterol, sitosterol, kaempferol, and (+)-catechin in B can regulate 47 targets and then act on the TNF signaling pathway, HIF-1 signaling pathway, IL-17 signaling pathway, MAPK signaling pathway, RA, and other pathways related to inflammation (Tables S2 and S3), which may be important potential biological mechanisms for B treatment of RA (Figure 7a).

Regarding network construction of the target pathway for the above ingredients and degree unDir, the results show that the average degree unDir was 7, while for kaempferol it was 19, the highest among the ingredients of B. AKT1 has the highest degree unDir, meaning it participated in most pathways (Table S3) and could interact with kaempferol. Therefore, molecular docking of the two combinations was conducted, and paeoniflorin was a famous compound in B marked as a key indicator for detecting B quality by the Chinese pharmacopoeia. Therefore, the binding analysis of action targets was also carried out in this study. The results show that kaempferol can bind to the residue LYS-67 of the most relevant target AKT (PDB ID: 4GAH) with a binding energy of −3.24 kcal/mol (Figure 7c), while for paeoniflorin and the target TNF (PDB ID: 1DU3), the docking binding energy of the residue CLY-245 was −0.99 kcal/mol (Figure 7b).

Palbinone, paeoniflorin, mairin, beta-sitosterol, sitosterol, kaempferol, and (+)-catechin can bind to the corresponding targets in different numbers, and they are sorted according to the degree unDir of binding from high to low as kaempferol, beta- sitosterol, (+)-catechin, paeoniflorin, mairin, sitosterol, and palbinone.

## 4. Discussion

The chronic etiology of the AIA rat model is comparable to the clinical manifestations of RA, making it a classic and popular choice for pharmacological research and experimental evaluation. Synovitis and cartilage degeneration eventually develop from the initial inflammation [18,19]. Although the pathogenesis of RA is unclear, the majority of researchers concentrate on omics studies with inflammatory responses and immunological abnormalities. CFA was injected into the rats’ roots to construct an AIA animal model comparable to RA. The spleen and thymus, which are both crucial immune organs, have lesions, spleen tissue edema, intercellular spaces mashed with inflammatory cells, thymus atrophy, and cell gap narrowing. The rat ankle X-ray data showed that there was severe tissue edema in the joints of the model rats. Neutrophils and macrophages were prevalent in the pathophysiology of the synovial tissue. After 28 days of oral treatment with B, the aforementioned occurrences were diminished, and B had a positive therapeutic effect.

Metabolomics technology can collect batch sample data information in a short time, especially with the high sensitivity of MS as the instrument to help in the discovery and identification of metabolites. The current metabolomic analysis strategy is widely employed in RA metabolic mechanism research and biomarker development [20,21,22,23]. In this study, UPLC-Q/TOF-MS was used to perform an untargeted metabolomics analysis of AIA rat urine to explore the overall metabolic changes and characterize the biomarkers. As a result, 29 biomarkers with a VIP > 1 and normalized abundance significance of *p* < 0.05 were found (Table S1), which participated in 15 metabolic pathways. It is worth noting that the AIA rats reversed the levels of 12 metabolites after 28 days of oral administration of B, including 1H-indole-3-carboxaldehyde, 4,6-dihydroxyquinoline, 7-methyladenine, lndoleacrylic acid, N2-succinyl-l-ornithine, phenyl pyruvic acid, porphobilinogen, semilepidinoside B, thymidine, urocanic acid, 3,4-dihydroxyhydrocinnamic acid, and sepacic acid. In addition to the above significantly reversed metabolites, after 28 days of oral administration of B, a concentration reversal occurred in the urine of the AIA rats for 13-L-hydroperoxylinoleic acid, 9-cis-retinoic acid, betaine, and pyruvic acid, but there was no significance, which may have been caused by a short cycle of medication. Other unresponsive metabolisms may not be the pharmacodynamic pathways for B to the AIA rats.

RA is a chronic and progressive inflammatory disease closely related to the environment and autoimmunity. Previous reports have shown that there are inflammatory factors in the synovial tissue fluid, serum, and urine of RA patients and biological models, and some metabolic disorders are mainly related to energy metabolism, lipid metabolism, and amino acid metabolism. The phenotypes are joint swelling and synovial membrane inflammation, bone destruction, pain, and mobility impairment [24,25]. Phenylalanine is an essential amino acid and has important physiological effects, and its levels in the synovium are related to the expression of IL-1β and IL-8 [25]. Metabolic abnormalities of phenylalanine were revealed in the patient-based metabolomics study samples. Ouyang’s study was based on ^1^H NMR [26], while Zhou applied GC-MS [27] and Urbaniak applied LC-MS [28] to illustrate low levels of phenylalanine in the patient’s serum, and Li applied LC-MS to find high levels of phenylalanine in the serum of patients [29]. Although our study did not directly observe the statistical differences between the control group and model group of phenylalanine, it was found that B can significantly reverse the levels of phenyl pyruvic acid in the phenylalanine metabolic pathway, while high levels of phenyl pyruvic acid may also affect phenylalanine production through negative feedback from enzyme 1.4.1.20 [30]. It is worth mentioning that arginine and proline metabolism crosstalk was also found in the serum of patients with abnormal phenylalanine metabolism, which was specifically manifested by a significant decrease in the level of arginine [28]. Arginine is considered an important amino acid in nutrition, nitric oxide synthase regulation, or nerve transmission in the central nervous system, which affects the interference response by enhancing nitric oxide synthesis and in turn produces the goose inflammatory factors TNF and interleukin, and the serum arginine concentration decreases in the immune response [31,32,33]. Similar to previous studies, N2-succinyl-L-ornithine in this study responded abnormally in the arginine and proline pathway, but B significantly regulated this metabolic condition (Figure 6 and Table S1). This study shows that in abnormal histidine metabolism, the significant decrease in the level of urocanic acid in the urine of AIA rats may be the cause of histamine conversion, and a decrease in the histamine level is a disadvantage to RA patients [34]. The inflammatory environment of patients with RA stimulates the breakdown of histidine into histamine and stimulates the proliferation of chondrocytes [35]. For the results of this study, in the same 1-methylhistidine and urocanic acid which belong to histidine metabolism, B significantly reversed the level of urocanic acid but did not regulate 1-methylhistidine or its trend. Clonal expansion of autoreactive T cells may cause RA onset, and pyrimidine metabolism of dihydroorotate dehydrogenase is the source pathogenic mechanism, so blocking pyrimidine metabolism may block lymphocyte proliferation and inhibit disease progression [36,37]. This study shows that B reduces the level of thymidine in the urine of AIA rats to regulate pyrimidine metabolism, which may protect against the inflammatory injury and lesion formation caused by CFA.

Predictive analysis of the ingredients in B by network pharmacology showed that the TNF signaling pathway, HIF-1 signaling pathway, IL-17 signaling pathway, osteoclast differentiation, and MAPK signaling pathway were the main pathways of action. These pathways harm the disease of RA. After their activation, the production of independent or interoperable inflammatory factors including TNF and interleukin and hypoxia-inducing factors, triggering inflammatory storms to exacerbate the immune response, promote synovial cell proliferation and fibrosis, gradually forming vascular fins and damaged cartilage and promoting the development of RA [38,39,40,41]. Fortunately, the ingredients in B may modulate the pathway and have the potential for prevention. The total glucosides of paeony were extracted from the root of *Paeonia lactiflora* Pall, where more than 90% of the composition is paeoniflorin, which is the active ingredient against RA according to previous reports [7,42]. Additionally, the anti-inflammatory abilities of palbinone [9], beta-sitosterol [43], kaempferol [44], and (+)-catechin [45] have been verified in experimental conditions, and the authors believe there is a considerable prospect for the development of anti-RA lead compounds.

## 5. Conclusions

This study examined the anti-RA effects of B and investigated the metabolic processes in experimental rats with MS. B inhibited CFA-induced claw swelling in rats with RA and alleviated the pathological conditions of the spleen and thymus. The model rat’s disordered metabolism, which included histidine metabolism, phenylalanine metabolism, arginine and proline metabolism, and pyrimidine metabolism, was corrected after 28 days of continuous administration. Paeoniflorin, palbinone, beta-sitosterol, kaempferol, and catechin may be important components. In sum, this research focused on the experimental data of B’s drug development and screening projects and conducted preliminary pharmacodynamic and pharmacological studies on B.

## Data Availability

The data that support the findings of this study are available from the corresponding author upon reasonable request.

## Supplementary Materials

The following supporting information can be downloaded at: https://www.mdpi.com/article/10.3390/molecules27207014/s1, Table S1: Identification and pathways of potential biomarkers of RA based on urine metabolic profiling; Table S2: The chemical constituents of *Radix Paeoniae Rubra* in TCMSP 2.3 database effect on RA related targets; Table S3: Target predicts the top 25 KEGG pathways of significance.

## Sample Availability

Samples of the compounds are not available from the authors.

## Abbreviations

B: *Paeoniae Radix Alba*; AIA: adjuvant-induced arthritis; RA: rheumatoid arthritis; CFA: Freund’s complete adjuvant; AI: arthritis index; UPLC-Q/TOF-MS: ultra-performance liquid chromatography quadrupole time of flight mass spectrometry; PCA: principal ingredient analysis; OPLS-DA: orthogonal partial least squares discriminant analysis; VIP: variable importance in projection.
