# Supplementary material for: High-Throughput Metabolomics Integrated Network Pharmacology Reveals the Underlying Mechanism of Paeoniae Radix Alba Treating Rheumatoid Arthritis
Source: Molecules. 2022 Oct 18;27(20):7014. doi: 10.3390/molecules27207014 (PMC9609690; doi:10.3390/molecules27207014)
Supplement: Supplementary file 1 [file molecules-27-07014-s001.zip › molecules-1944113-supplementary.pdf]

Table S1. Identification and pathways of potential biomarkers of RA based on urine metabolic profiling

No.	Compound	m/z	Mass Error (ppm)	Compound ID	Description	Adducts	Formula	Pathway	Con vs. Mod	B vs. Mod
1	9.80_313.2346m/z	313.23	4.32	HMDB0003871	13-L-Hydroperoxylinoleic acid	M+H	$C_{18}H_{32}O_4$	Linoleic acid metabolism	*	-
2	4.28_146.0606m/z	146.06	4.65	HMDB0029737	1H-Indole-3-carboxaldehyde	M+H	C_9H_7NO	-	***	#
3	0.89_170.0929m/z	170.09	9.56	HMDB0000001	1-Methylhistidine	M+H	$C_7H_{11}N_3O_2$	Histidine metabolism	*	-
4	3.90_162.0557m/z	162.06	9.21	HMDB0004077	4,6-Dihydroxyquinoline	M+H	$C_9H_7NO_2$	Tryptophan metabolism	*	##
5	1.98_150.0780m/z	150.08	4.48	HMDB0011614	7-Methyladenine	M+H	$C_6H_7N_5$	-	***	###
6	9.51_279.2300m/z	279.23	4.32	HMDB0001388	Alpha-Linolenic acid	M+H	$C_{18}H_{30}O_2$	alpha-Linolenic acid metabolism, Biosynthesis of unsaturated fatty acids	***	-
7	0.52_118.0870m/z	118.09	5.79	HMDB0000043	Betaine	M+H	$C_5H_{11}NO_2$	Glycine, serine and threonine metabolism	**	-
8	10.21_450.3250m/z	450.33	4.70	HMDB0006898	Chenodeoxyglycocholic acid	M+H	$C_{26}H_{43}NO_5$	-	***	-
9	3.91_188.0715m/z	188.07	9.21	HMDB0000734	Indoleacrylic acid	M+H	$C_{11}H_9NO_2$	-	**	#
10	1.77_233.1138m/z	233.11	4.06	HMDB0001199	N2-Succinyl-L-ornithine	M+H	$C_9H_{16}N_2O_5$	Arginine and proline metabolism	***	###
11	5.28_283.2649m/z	283.26	-5.12	HMDB0000207	Oleic acid	M+H	$C_{18}H_{34}O_2$	Biosynthesis of unsaturated fatty acids	*	-
12	3.79_165.0554m/z	165.06	5.02	HMDB0000205	Phenylpyruvic acid	M+H	$C_9H_8O_3$	Phenylalanine metabolism, Phenylalanine, tyrosine and tryptophan biosynthesis	**	###
13	3.46_227.1034m/z	227.10	5.02	HMDB0000245	Porphobilinogen	M+H	$C_{10}H_{14}N_2O_4$	Porphyrin and chlorophyll metabolism	**	##
14	0.65_144.1029m/z	144.10	2.31	HMDB0004827	Proline betaine	M+H	$C_7H_{13}NO_2$	-	***	-
15	3.14_367.1510m/z	367.15	1.96	HMDB0033108	Semilepidinoside B	M+H	$C_{17}H_{22}N_2O_7$	-	***	#
16	0.71_243.0979m/z	243.10	4.34	HMDB0000273	Thymidine	M+H	$C_{10}H_{14}N_2O_5$	Pyrimidine metabolism	***	###
17	1.17_139.0509m/z	139.05	6.49	HMDB0000301	Urocanic acid	M+H	$C_6H_6N_2O_2$	Histidine metabolism	***	#
18	7.61_301.1819m/z	301.18	7.45	HMDB0000405	2-Methoxyestradiol	M-H	$C_{19}H_{26}O_3$	Steroid hormone biosynthesis	***	-
19	4.50_181.0498m/z	181.05	-4.66	HMDB0000423	3,4-Dihydroxyhydrocinnamic acid	M-H	$C_9H_{10}O_4$	Tyrosine metabolism	*	###
20	3.40_148.0397m/z	148.04	-5.15	HMDB0004058	5,6-Dihydroxyindole	M-H	$C_8H_7NO_2$	Tyrosine metabolism	**	-
21	10.11_295.2265m/z	295.23	-4.28	HMDB0004701	9,10-Epoxyoctadecenoic acid	M-H	$C_{18}H_{32}O_3$	Linoleic acid metabolism	**	-
22	9.48_329.2325m/z	329.23	-4.28	HMDB0004708	9,12,13-TriHOME	M-H	$C_{18}H_{34}O_5$	Linoleic acid metabolism	*	-
23	9.93_299.2022m/z	299.20	-4.28	HMDB0002369	9-cis-Retinoic acid	M-H	$C_{20}H_{28}O_2$	Retinol metabolism	*	-
24	0.60_179.0550m/z	179.05	-2.48	HMDB0000122	D-Glucose	M-H	$C_6H_{12}O_6$	Glycolysis / Gluconeogenesis, Pentose phosphate pathway, Galactose metabolism, Starch and sucrose metabolism, Amino sugar and nucleotide sugar metabolism	*	-
25	10.77_301.2166m/z	301.22	-2.50	HMDB0001999	Eicosapentaenoic acid	M-H	$C_{20}H_{30}O_2$	Biosynthesis of unsaturated fatty acids	**	-
26	3.28_178.0502m/z	178.05	-5.15	HMDB0000714	Hippuric acid	M-H	$C_9H_9NO_3$	Phenylalanine metabolism	*	-
27	1.27_87.0080m/z	87.01	-5.15	HMDB0000243	Pyruvic acid	M-H	$C_3H_4O_3$	Glycolysis / Gluconeogenesis, TCA cycle,	*	-

								Pentose phosphate pathway, Pentose and glucuronate interconversions, Ascorbate and aldarate metabolism		
28	5.52_201.1121m/z	201.11	-2.53	HMDB0000792	Sebacic acid	M-H	$C_{10}H_{18}O_4$	-	**	##
29	0.47_124.0067m/z	124.01	-2.48	HMDB0000251	Taurine	M-H	$C_2H_7NO_3S$	Primary bile acid biosynthesis, Taurine and hypotaurine metabolism, Sulfur metabolism	**	-

Statistical results of the normalized abundance of biomarkers: Control vs. Model, $^{*}p<0.05$, $^{**}p<0.01$, $^{***}p<0.001$; the normalized abundance of biomarkers reversed after administration, B vs. Model, $^{\#}p<0.05$, $^{\#\#}p<0.01$, $^{\#\#\#}p<0.001$.

Table S2 The chemical constituents of *Radix Paeoniae Rubra* in TCMSP 2.3 database effect on RA related targets

Name	CAS	MW	OB (%)	DL	Target
palbinone	139954-00-0	358.52	43.56	0.53	PGR
paeoniflorin	23180-57-6	480.51	53.87	0.79	TNF
mairin	472-15-1	456.78	55.38	0.78	PGR
beta-sitosterol	83-46-5	414.79	36.91	0.75	PGR, PTGS1, PTGS2, PIK3CG, CHRM3, PDE3A, HTR2A, ADRA1A, ADRB2, CHRNA7, BCL2, JUN, CASP3, CASP8, PRKCA, TGFB1, PON1
sitosterol	149-91-7	414.79	36.91	0.75	PGR
kaempferol	520-18-3	286.25	41.88	0.24	AHR, CYP3A4, HMOX1, NOS3, PPARG, PPARG, PTGS2, AKT1, F2, TNF, VEGFA, AHSA1, BCL2, CASP3, CYP1A1, CYP1A2, DPP4, SELE, GSTM1, GSTM2, GSTP1, INSR, ICAM1, MMP1, NOS2, NR1I2, NR1I3, PIK3CG, PGR, PTGS1, STAT1, SLC6A2, JUN, RELA, VCAM1
catechin	154-23-4	290.29	54.83	0.24	ESR1, PTGS2, PTGS1, RXRA

Table S3 Target predicts the top 25 KEGG pathways of significance

term ID	term description	matching proteins in network
hsa04933	AGE-RAGE signaling pathway in diabetic complications	TGFB1, ICAM1, VCAM1, NOS3, CASP3, SELE, STAT1, JUN, BCL2, RELA, TNF, PRKCA, AKT1, VEGFA
hsa05200	Pathways in cancer	HMOX1, TGFB1, GSTM2, PPARG, CASP3, GSTM1, MMP1, NOS2, CASP8, STAT1, PTGS2, JUN, BCL2, GSTP1, RELA, ESR1, PRKCA, RXRA, AKT1, VEGFA
hsa05418	Fluid shear stress and atherosclerosis	HMOX1, GSTM2, ICAM1, VCAM1, NOS3, GSTM1, SELE, JUN, BCL2, GSTP1, RELA, TNF, AKT1, VEGFA
hsa04668	TNF signaling pathway	ICAM1, VCAM1, CASP3, SELE, CASP8, PTGS2, JUN, RELA, TNF, AKT1
hsa05145	Toxoplasmosis	TGFB1, CASP3, NOS2, CASP8, PIK3CG, STAT1, BCL2, RELA, TNF, AKT1
hsa05161	Hepatitis B	TGFB1, CASP3, CASP8, STAT1, JUN, BCL2, RELA, TNF, PRKCA, AKT1
hsa04066	HIF-1 signaling pathway	HMOX1, NOS3, INSR, NOS2, BCL2, RELA, PRKCA, AKT1, VEGFA
hsa05167	Kaposi's sarcoma-associated herpesvirus infection	ICAM1, CASP3, CASP8, PIK3CG, STAT1, PTGS2, JUN, RELA, AKT1, VEGFA
hsa05204	Chemical carcinogenesis	GSTM2, GSTM1, CYP3A4, CYP1A2, PTGS2, CYP1A1, GSTP1, CHRNA7
hsa04926	Relaxin signaling pathway	TGFB1, NOS3, MMP1, NOS2, JUN, RELA, PRKCA, AKT1, VEGFA
hsa04932	Non-alcoholic fatty liver disease (NAFLD)	TGFB1, INSR, CASP3, CASP8, JUN, RELA, TNF, RXRA, AKT1
hsa05152	Tuberculosis	TGFB1, CASP3, NOS2, CASP8, STAT1, BCL2, RELA, TNF, AKT1
hsa01524	Platinum drug resistance	GSTM2, CASP3, GSTM1, CASP8, BCL2, GSTP1, AKT1
hsa05140	Leishmaniasis	TGFB1, NOS2, STAT1, PTGS2, JUN, RELA, TNF
hsa04657	IL-17 signaling pathway	CASP3, MMP1, CASP8, PTGS2, JUN, RELA, TNF
hsa05222	Small cell lung cancer	CASP3, NOS2, PTGS2, BCL2, RELA, RXRA, AKT1
hsa05142	Chagas disease (American trypanosomiasis)	TGFB1, NOS2, CASP8, JUN, RELA, TNF, AKT1
hsa04020	Calcium signaling pathway	CHRM3, NOS3, ADRB2, NOS2, ADRA1A, CHRNA7, PRKCA, HTR2A
hsa04380	Osteoclast differentiation	TGFB1, PPARG, STAT1, JUN, RELA, TNF, AKT1
hsa00980	Metabolism of xenobiotics by cytochrome P450	GSTM2, GSTM1, CYP3A4, CYP1A2, CYP1A1, GSTP1
hsa04010	MAPK signaling pathway	TGFB1, INSR, CASP3, JUN, RELA, TNF, PRKCA, AKT1, VEGFA
hsa05143	African trypanosomiasis	ICAM1, VCAM1, SELE, TNF, PRKCA
hsa04210	Apoptosis	CASP3, CASP8, JUN, BCL2, RELA, TNF, AKT1
hsa05323	Rheumatoid arthritis	TGFB1, ICAM1, MMP1, JUN, TNF, VEGFA
hsa04022	cGMP-PKG signaling pathway	NOS3, INSR, ADRB2, PDE3A, PIK3CG, ADRA1A, AKT1

Note: A pathway for the active involvement of AKT1.
